# Colorectal cancer liver metastases within the central and peripheral segments: Parenchymal sparing surgery adaptation

**DOI:** 10.1016/j.amsu.2020.07.052

**Published:** 2020-08-14

**Authors:** A.A. Burlaka, A.V. Paliichuk, O.I. Iatsyna, О.О. Kolesnik

**Affiliations:** a Colorectal Cancer Department of National Cancer Institute, Ukraine; b Medical Centre “Omega-Kyiv”, Ukraine; c Medical Director of National Cancer Institute, Ukraine; d Main Researcher of Colorectal Cancer Department of National Cancer Institute, Ukraine

**Keywords:** Parenchymal sparing liver surgery, R1 vascular, Resection margin, Colorectal cancer liver metastases, Hard to reach liver cites

## Abstract

**Background:**

The debate over the surgical strategy optimization in colorectal cancer patients with liver metastases (mCRC) has been ongoing in the last 20 years. However, parenchyma sparing surgery (PPS) in cases of hard to reach liver cites (HTRLC) remain to be controversial.

**Methods:**

A prospective analysis of 185 mCRC patients performed who were devided in two groups depending by predominant liver cite localization. Peripherally localized metastases (PLM) (n = 107) (S2, S3, S6, S7, Spiegel lobe and subcapsular area 1–2 cm below the liver surface). Group 2 included those with metastases localized in HTRLC (n = 78) - metastatic lesions of the “right venous core”, portal and caval hilum, paracaval part of S1, “deep” parenchyma cites of S5, S8 and S4.

**Results:**

In 26 (33,3%) and 32 (29,9%) patients of HTRLC and PLM, respectively, performed one liver re-resection (0,62). In HTRLC group 2 and more re-resection were performed in 7 (8,9%) cases while in PLM in 11 (10,3%), p = 0,76. Postoperative major morbidity was 24,4%, 21,8% (p = 0,15) and mortality 8,9%, 4,6% for HTRLC and PLM groups, respectively. R1v principles were implemented in 24 (30,7%) cases with centrally located metastases and in only 6 cases (5.6%) with peripheral localized metastases (p = 0,001). Cumulative 3-year disease-free survival (DSF) for PLM and HTRLC groups was 63% and 41% (p = 0,008). DFS for R1v (n = 24) and R0 (54) cochorts in HTRLC group was 33% and 43%, respeсtively (p = 0,44).

**Conclusions:**

Principles of the PPS tactic provides an adequate removal of metastatic lesions in hard to reach liver cites allowing to maintain organ functions and increases the feasibility of the repeated liver resections in case of the initial disease progression.

## Highlights

• The parenchymal preserving surgery allows to remove adequately metastatic lesions in “hard to reach liver cites”.
• Сumulative 3-year disease-free survival between groups PLM and HTRLC was was in total 63% and 41%, respectively (p=0,008).
• R1v detachment in combination with modern chemotherapy may be an effective method to reduce the cohort of unresectable cases.

## 1 Introduction

The debate over the feasibility of performing a wide resection margin in liver resections in patients with colorectal cancer metastases (mCRC) has been ongoing in the last 20 years. The 1-cm margin tactic feasibility explained by the results of several leading centers, which predicted significantly worse survival in patients with a smaller margin [1]. For the last 10 years these authors continued to publish evidence of the expediency of a wide deviation from the edge of the metastatic lesion, citing that a 1-cm margin allows to improve long-term outcomes [2].

Almost at the same time a number of specialized surgical centers began to defend the surgical practice which requires a 1-mm resection margin tactic. Recently, the same authors published data that R1 has less prognostic value as compared to the biological features of the primary tumor and its metastases [3,4]. This variety of published evidence has led to an active search for the only right solution that continues to current days. It should be mentioned that most publications on the results of resections of multiple metastases are similar in their approach to retreat. At the same time, experts claim that they used the minimal margin of retreat to maximize preservation of the liver parenchyma (PPS). Moreover, the tactic of single-stage ultrasound-controlled parenchymal preservation of the liver, an alternative tactic for bi-lobar metastatic lesions of the mCRC, which involves parenchymal preservation of all metastatic lesions in the liver at one stage [6] are actively implemented. The latter approach is also considered to be an adapted “cherry-picking surgery” technique used to remove sub-capsular liver metastases [7]. Torzilli et al. have expanded the indications for ultrasound-controlled parenchymal preservation of the liver and adapted this technology to remove deep located metastatic lesions in the liver [8,14].

The latest consensus on the strategy for the surgical treatment of colorectal cancer metastases published in 2016 states clearly that a ≥1 mm margin is sufficient and safe [9]. However, the results of the parenchyma sparing strategy in cases of hard to reach liver cites (HTRLC) mCRC lesions localization (“right venous core”, portal and caval hilum, segment 1 paracaval part and others) remain to be unclear.

## 2 Material and methods

**Fig. 1:**
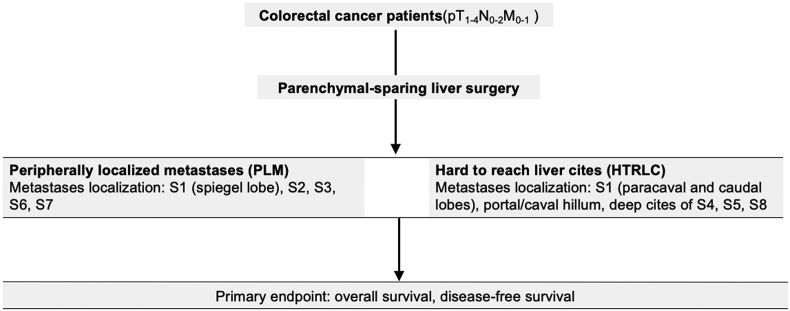
Study design.

A prospective analysis of the results of surgical and combined treatment of mCRC patients had been done. Patients (n = 185) with mCRC (pT_1-4_N_0-2_M_0-1_ colorectal cancer and pT_1-3_N_0-2_M_0-1_ rectal cancer) treated in National Cancer Institute during the period January 2015–July 2020 were enrolled into this study (Fig. 1).

Depending on the localization of metastatic lesions all patients were divided into two groups. First group included patients with peripherally localized metastases (PLM) (n = 107) and predominant allocation in left lateral section (S2, S3) and posterior section (S6, S7), Spiegel lobe of S1 and subcapsular area 1–2 cm below the liver surface. Group 2 included those with metastases localized in HTRLC (n = 78) - metastatic lesions of the “right venous core”, portal and caval hilum, paracaval part of S1, “deep” parenchyma cites of S5, S8 and S4.

Inclusion criteria was mCRC patients with ≥1 liver metastases, considered resectable (possibility of ≥30% parenchyma preservation). Exclusion criteria stated for patients who have more than 3 lung metastases, and/or peritoneal carcinomatosis. Primary outcome assessed with perioperative morbidity and mortality according to the Dindo-Clavien classification. Secondary outcome measured by analyzing an overall and disease-free survival. The rate of local recurrence after a minimal follow-up of 4 months, the long-term follow-up, analyzing the overall survival (survival after surgery), time to recurrence (survival without recurrence). Surgical technique included crash-clamping technique with resection margin size ≥1 mm. In possible cases a tactic of “vascular detachment” (R1v) has been used. Ischemia technique included classical and selective Pringle maneuver (20 min - ischemia, 5 min - reperfusion). All operations were accompanied by intraoperative ultrasound navigation.

Complication data were collected from the medical record. Major complications were defined either as requiring intensive care unit stay, treatment by an interventional radiologist, or reoperation, or as resulting in death. Complications stemming from dysfunction of the liver or biliary system were defined as liver related.

## 3 Statistical analysis

Survival analyses was done using Kaplan–Meier method, the log rank test to compare outcomes between two groups. A *t*-test was used to compare quantitative variables between groups if the distribution was parametric; ANOVA followed by the post hoc test and nonparametric test (Mann-Whitney *U* test) were used to test significance of differences. Statistical significance was determined as P < 0.05. Values are expressed as median ± min. and max. Statistical analyses performed using IBM SPSS® version 25.0 (IBM, Armonk, New York, USA).

## 4 Results

185 patients with mCRC who underwent liver resections were included into the study. Study groups show no significant difference in number of the removed metastatic lesions - 6 (1–16) and 7 (2–19) for HTRLC and PLM, respectively (p = 0,16).

The only difference was found in cochorts of patients that have been diagnosed from 2 to 5 metastases (30,7% and 47,6%, respectively) (p = 0,02). Solitary metastatic lesions were detected in 10 (12,8%) patients from HTRLC group and in 14 (13,1%) from PLM locations, p = 0,95. There were no difference in subgroups with more than 5 metastases (Table 1). Bilobar metastases were found in both groups with similar frequency - in 29 (37,2%) and 31 (28,9%) patients for HTRLC and PLM, categorically (p = 0,24). Moreover, volumetric data shows that the median value of the metastatic lesions volume was similar in PLM patients (127 cm^3^) and HTRLC (95 cm^3^), p = 0,1. In ½ cases of HTRLC group liver metastases were found to have synchronous status, while in PLM group it was in 59,8%.

**Table 1:** Surgery and tumor data characteristics.

Values	HTRLC (n = 78), (%)	PLM (n = 107), (%)	p value
Number of the resected lesions, median (min-max)	6 (1–16)	7 (2–19)	0,16
Distribution based on number of metastatic lesions			
1	10 (12,8)	14 (13,1)	0,95
2-5	24 (30,7)	51 (47,6)	**0,02**
6-10	19 (24,4)	16 (14,9)	0,1
11-15	23 (29,5)	21 (19,6)	0,2
≥ 15	2 (2,5)	5 (4,6)	0,46
Bi-lobar liver metastases	29 (37,2)	31 (28,9)	0,24
Synchronous status of liver metastases	39 (50)	64 (59,8)	0,18
Simultaneous surgery colon/rectal and liver	14 (17,9)	19 (17,7)	0,97
Left-sided/right-sided primary tumor localization	64(82,1)/14(17,9)	93(86,9)/14(13,1)	0,36
Primary tumor surgery: laparoscopic/open	57(73,1)/21(26,9)	78(72,8)/29(27,1)	0,97
R1v resection margin	24 (30,7)	6 (5,6)	**0,001**
Re-resections			
1	26 (33,3)	32 (29,9)	0,62
2 and more	7 (8,9)	11 (10,3)	0,76
Major liver surgery	5 (6,4)	14 (13,1)	0,14
Surgical incisions:			
Upper midline laparotomy	1 (1,3)	5 (4,6)	0,2
J-shaped right laparotomy	68 (87,2)	91 (85,1)	0,6
Total laparotomy	9 (11,5)	11 (10,2)	0,7
The volume of the surgically removed tumor tissue (сm^3^), median (min-max)^a^	95 (12–236)	127 (3–274)	0,1

a - metastatic lesions volumetry was used.

Left-sided and right-sided primary tumors made up the majority in groups and distributed as 64 (82,1%) and 93 (86,9%) in HTRLC and PLM respectively (p = 0,36). Primary tumor surgery was performed laparoscopically in 73,1% and 72,8% for HTRLC and PLM groups,. Simultaneous surgery with one stage primary colon or rectal resection and liver PPS used for both, HTRLC (17,9%) and PLM (17,7%) patients, p = 0,97.

Standard colon cancer surgery included a complete mesocolonectomy within the anatomical-fascial embryonic compartments and central ligation of colon vessels (Complete Mesocolic Excision with Central Vascular Ligation). Rectal surgery involved total or partial mesorectumectomy, lymph mode dissection in colon and rectal cancer volume was D3. 145 (78,4%) patients were operated in the department of Colorectal cancer of National Cancer Institute, Kyiv, Ukraine. Chemo- and radiotherapy performed in accordance with latest NCCN recommendations [15].

In total, 141 of the 185 patients developed recurrence after curative resection, and 76 (53,9%) of these underwent repeat resection for recurrence. In 26 (33,3%) and 32 (29,9%) patients for HTRLC and PLM, respectively one liver re-resection was completed (0,62). In group of completed one re-resection, 55 (72,4%) had diseases re-recurrence and 18 (32,7%) of these patients underwent a second two or more repeated resection. In HTRLC group 2 and more re-resection were performed in 7 (8,9%) cases while in PLM in 11 (10,3%), p = 0,76.

J-shaped right laparotomy used mostly for HTRLC (87,2%) and PLM (85,1%), respectively (p = 0,28), whereas total and upper midline laparatomy accomplished less frequently (Table 1). HTRLC resection using the liver parenchyma preservation principles required more frequent use of the R1v approach. It was implemented in 24 (30,7%) cases with centrally located metastases and in only 6 cases (5.6%) with peripheral localized metastases (p = 0,001). The duration of surgery didn't correlate with metastatic lesion location with median 306 min and 285 min values for HTRLC group and PLM group, respectively (p = 0,08). Major liver surgery (resection of 3 and more anatomical liver segments) done in 5 cases (6,4%) and 14 cases (13,1%) for HTRLC and PLM group, respectively (p = 0,14).

Postoperative major morbidity was 19 (24,4%) and 17 (21,8%), respectively, for the comparison groups (p = 0,15). There weren't differences in comparison groups by liver-specific morbidity, 8,9% and 4,6% for HTRLC and PLM groups, respectively. In a group with centrally located metastases acute liver failure was never higher than grade A (5 (6,4%)). In PLM group 8 (7.5%) cases of acute liver failure grade A and 1 (0.9%) grade B were diagnosed. Most frequent liver specific major complications were bile leakage and biloma. The median duration of warm ischemia during parenchyma transection was significantly higher in patients who underwent surgery for an HTRLC metastases – 39 ± 25,8 min as compared to PLM group - 15 ± 18,05 min (p < 0,001). Central PPS resections of the liver weren't associated with higher blood loss or blood units transfusion (Table 2). Іnterestingly, mortality rate in 30-day postoperative period was reported only in HTRLC group (1,3%), induced by mesenteric thrombosis at 5th postoperative day. 90-day mortality was similar in both groups 1,3% and 0,9% for HTRLC and PLM, respectively.

**Table 2:** Operative and perioperative data.

Value	HTRLC (n = 78), (%)	PLM(n = 107), (%)	p value
Blood units transfusion, median (min-max)	0 (0–4)	0 (0–2)	0,2
Surgery duration (min), median (min-max)	306 (41–520)	285 (35–735)	0,08
Liver warm ischemia duration, min. ± SD	39 ± 25,8	15 ± 18,05	**0,001**
Overall morbidity	30 (38,5)	44 (41,1)	0,6
Major morbidity	19 (24,4)	17 (21,8)	0,15
Liver-specific morbidity	7 (8,9)	5 (4,6)	0,3
30-day mortality	1 (1,3)	–	
90-day mortality	1 (1,3)	1 (0,9)	

**Fig. 2:**
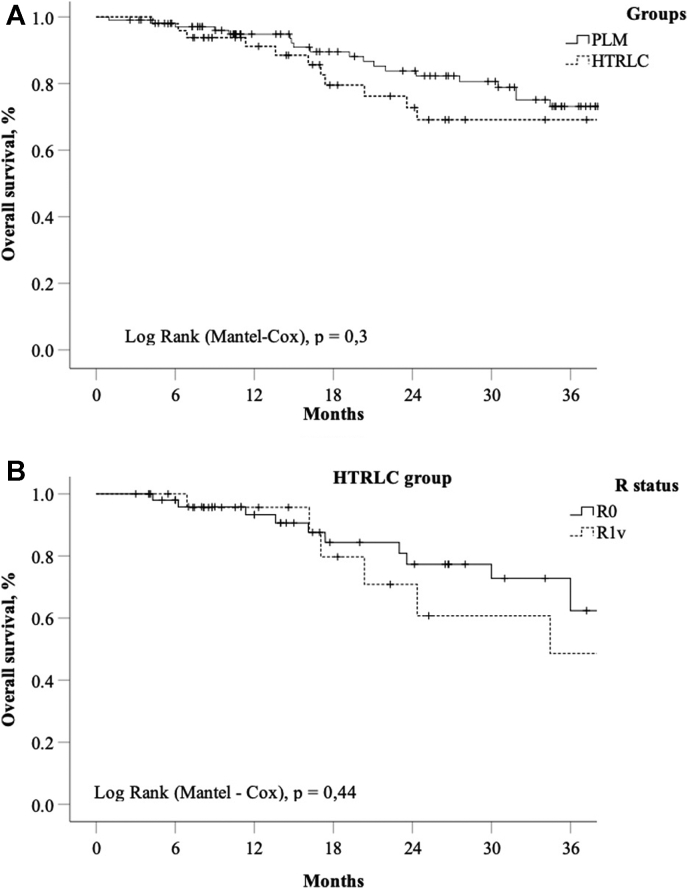
A, Kaplan-Meier plot of overall survival for 185 mCRC patients undergoing in PLM and HTRLC groups. B, Kaplan-Meier plot of overall survival for 78 mCRC patients of HTRLC group with liver resection margin stratification (R0/R1v).

Fig. 1A demonstrates the overall cumulative survival in presented groups of patients (n = 185). The estimate 3-year overall survival was 74%, 66% for the HTRLC and PLM, respectively, p = 0,3 (Fig. 2A). Also 3-year overall cumulative survival for all included 185 patients was 68%. As was mentioned previously, the R1v approach used in 24 patients of HTRLC group and only in 6 of PLM. That is why we demonstrate survival in HTRLC group using resection margin status as stratification variable. Fig. 2B demonstrates the overall 3-year cumulative survival rates (73% and 48%) in cohorts with R0 (n = 54) and R1v (n = 24) liver resection margins, respectively (p = 0,44).

**Fig. 3:**
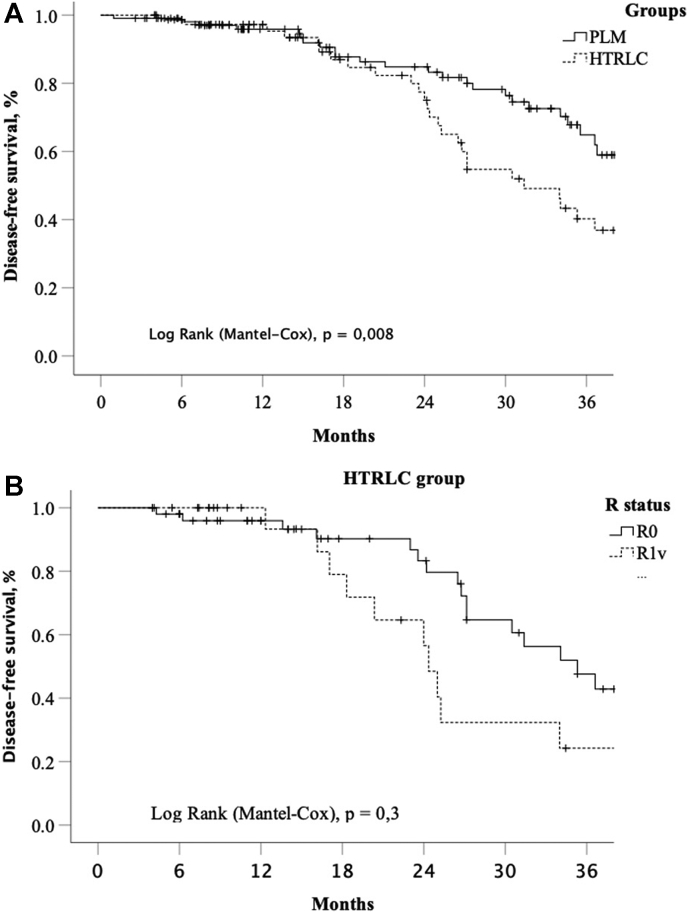
A, Kaplan-Meier plot of disease-free survival for 185 mCRC patients undergoing in PLM and HTRLC groups. B, Kaplan-Meier plot of disease-free survival for 178 mCRC patients of HTRLC group with liver resection margin stratification (R0/R1v).

Furthermore, the difference in cumulative 3-year disease-free survival between groups PLM and HTRLC was significant (p = 0,008) and was in total 63% and 41%, respectively (Fig. 3A). Also the analysis of R1v (n = 24) and R0 (54) subgroups of HTRLC demonstrates the 3-year disease-free survival on level 33% and 43%, respeсtively (p = 0,44), (Fig. 3B).

## 5 Discussion

Surgical removal of the primary tumor and all areas affected by distant metastases is a priority approach in the survival rate of such patients, despite the constant development of systemic chemotherapy. More than 50% of patients with liver resection due to metastatic liver disease in anamnesis have a risk of recurrent metastatic disease, which usually requires combined treatment with line 2 chemotherapy and resection. The principle of a wide margin liver resection (1 cm) of hepatic metastatic lesions of CRC encourage surgeons to perform major liver surgery. This tactic in most cases leads to a high level of severe complications including acute liver failure in the early postoperative period and small liver syndrome in a later period, which occurs according to different data in 4–16% of cases [10]. Thus, the surgical strategy directly affects the possibility of adequate systemic treatment of such patients in the adjuvant mode. Moreover, the large resections tactic is accompanied by the challenge of R1 performing in 30% cases according to recent meta-analyzes [11].

From our point of view the approach that was set out above is an alternative in cases of centrally localized metastatic lesions (within the v.porta or v.cava “gates” of the liver). Classical surgical algorithm implies a wide retreat from the metastatic lesions that has close contacts without signs of true ingrowth into the first or second order of the Glissonean structures and into the orifice of the main hepatic veins. These conditions irreversibly forcing surgeons to perform “extended” liver resections. This tactic significantly increases the risk of acute liver failure because of the small future liver remnant volume and is the leading cause of death in patients after liver resection. Whereas artificial stimulation of hypertrophy (embolization or ligation of the traumatic vein) carries hidden risks of passive stimulation of the growth of disseminated micrometastases (minimal residual disease) [12]. Evidence for the possible initiation of the uncontrolled tumor growth based on chronic inflammation or artificial activation of hypertrophy mechanisms began to be published in the 80's and are still actively studied both experimentally and in the clinical studies (transplantology) [13].

Standard surgical strategy for mCRC patients with bilobar metastases is two-stage hepatectomy (TSH) which implies consecutive atypical resections in the left lobe, portal vein embolization (PVE) chemotherapy and right hemihepatectomy [16]. The main problem of TSH is “drop-out” of the 2nd surgical stage due to the uncontrolled tumor progression during chemotherapy and/or inadequate liver parenchyma hypertrophy [17]. The authors explain the feasibility of preliminary removal of metastases from perspective liver lobe by the method of prevention of uncontrolled growth under the conditions of artificial liver hypertrophy stimulation [18]. Small metastatic lesions (micrometastases) in the non-embolized lobe of the liver that were not detected by CT/MRI may be vizualized after PVE procedure due to the potential of the artificial environment that stimulates tumor growth [19]. Kokudo and others showed an increased index of proliferation in the tissue of adenocarcinoma metastases of mCRC in group of PVE [20]. The mean volume of metastatic tissue was significantly higher in the group of patients who underwent PVE by 20,8%, the authors performed volumetry 3 weeks after embolization. Moreover, in the group without PVE, there was no significant increase in metastatic tissue volume during the observation period.

Alternative technique was introduced in 2012, for mCRC patients initially seemed unresectable [21]. But some of the clinical trials showed higher tumor recurrence in the resected liver after the ALPPS rather than after TSH (8 of 8 versus 9 of 17 patients, respectively; P = 0,005) [22]. So far, the oncological effect of ALPPS remains poorly understood as no randomized trials have been conducted.

Latest data show that colorectal cancer tumor growth and spreading model suggest that all metastases are synchronous with the primary tumor and metachronous metastasis is thought to be a consequence of the failure of the immune surveillance of dormant micrometastatic lesions, which are present at an early stage of disease [23]. Also a diffusion of micrometastases and cancer dormancy of mCRC is the main argument against wide liver resection margin and/or extended liver surgery. Therefore, we believe that PPS tactic in combination with intraoperative ultrasound navigation, orientation in anatomy of vascular structures of 1–2 order due to CT/MRI modelling with 3D reconstructions and the use of “vascular detachment” will increase the chances of each patient to undergo the repeated resection.

As was mentioned previously, that only complete surgical resections of liver metastases are associated with an increased long-term survival in patients with mCRC. The aim of this study was to determine the oncological and surgical safety of PPS strategy with R1 vascular detachment approach in patients with HTRLC metastases location. The PPS tactics implied by us did not lead to an increase of liver specific major morbidity or mortality despite their technical complexity in HTRLC group. In group of patients with HTRLC resections longer warm ischemia applied due to the use of the Pringle maneuver. We found that more than a ½ of all patients could achieve one re-resection and 18 patients received 2 or more re-resections for recurrence. Oncological outcomes in this study are in line with world reference. Within the HTRLC and PLM groups 1/3 of all patients appear to be with bilobar metastatic disease (3 year disease-free cumulative survival rate was 41% and 63%, respectively). Cohort of patients from HTRLC group with resection margin status R1v demonstrated the 3-year disease-free survival at level 33%. Whereas the results of recent clinical trial with TSH and T-ALPPS strategies demonstrate the 3-year disease-free survival rates as followings - 9,5% and 11,1%, respectively [24].

## 6 Conclusions

It is proved that the use of parenchymal-sparing surgery tactic is safe and effective from the oncologic point of view for patients with colorectal cancer and bi-lobar metastatic liver disease. Principles of the parenchymal-sparing surgery tactic allows to remove adequately metastatic lesions in hard to reach liver cites allowing to maintain organ functions and increases the probability of the additional liver resections in case of the initial disease progression. R1 vascular tactic in combination with modern chemotherapy schemes may be an effective method to reduce the cohort of unresectable patients with bilobar metastatic liver disease resulted in satisfactory oncology outcome.

## Ethical approval

All patients in this study have signed ethical approval. The protocol proposed for signature was developed and approved by the Ethics Committee of the National Cancer Institute (№76 of 15.01.2015).

## Sources of funding

Ministry of health of Ukraine.

## Author contribution

Anton Burlaka – main idea, design and and paper writing.

Paliichuk Ariadna – CT and MRI data analysis, translating.

Oleksandr Iatsyna – supervisor.

Olena Kolesnik – supervisor.

## Registration of research studies

•Name of the registry: www.researchregistry.com.
•Unique Identifying number or registration ID: researchregistry5679.
• Hyperlink to your specific registration (must be publicly accessible and will be checked): https://www.researchregistry.com/browse-the-registry#home/?view_2_search=Anton%20Burlaka&view_2_page=1.

## Guarantor

O.I. Yatsyna, general director of National Cancer Institute.

https://unci.org.ua/yacina-oleksandr-ivanovich/.

## Declaration of competing interest

Authors don't have any conflict of interest.
